# Effect of Nanoemulsions of Betulinic Acid on the Development of Canine Mammary Tumors

**DOI:** 10.3390/vetsci12060522

**Published:** 2025-05-27

**Authors:** Zayra Yeretzi Amoros-Cerón, Juan Manuel Pinos-Rodríguez, Hugo Sergio García, Angélica Olivares-Muñoz, Isaac De Gasperin-López, Argel Flores-Primo

**Affiliations:** 1 Facultad de Medicina Veterinaria y Zootecnia, Universidad Veracruzana, Veracruz 91710, Mexico; zs22000202@estudiantes.uv.mx (Z.Y.A.-C.); aolivares@uv.mx (A.O.-M.); idegasperin@uv.mx (I.D.G.-L.); argflores@uv.mx (A.F.-P.); 2 Unidad de Investigación y Desarrollo en Alimentos, Tecnológico Nacional de México campus Veracruz (TecNM), Instituto Tecnológico de Veracruz, Veracruz 91897, Mexico; hugo.gg@veracruz.tecnm.mx

**Keywords:** betulinic acid, mammary gland, nanoemulsions

## Abstract

Mammary gland tumors in dogs are very common in clinical practice. Betulinic acid is currently a compound considered to have anticancer properties in human mammary tumors via nanoemulsions. In this study, betulinic acid nanoemulsions with a particle size of less than 300 nm were prepared. Biopsies were obtained from five female dogs with mammary tumors for histopathological analysis, confirming that two were tubular mammary carcinomas (MMTs, malignant) and three were complex mammary adenomas (BMTs, benign). The five female dogs were administered with a daily oral dose of nanoemulsion containing 5 mg/kg of betulinic acid for 30 days. Tumor size was measured every 7 days, and the response to treatment was assessed according to RECIST (Response Evaluation Criteria In Solid Tumors) standards. In one of the females with MMTs treated with the nanoemulsion, the tumor size was reduced by approximately 38%, while in the BMT female dogs, the nanoemulsion reduced the tumor size by 25.3%. It was concluded that oral administration of betulinic acid nanoemulsions reduced the size of canine mammary tumors. Experimental studies are still needed to further evaluate this preparation.

## Simple Summary

Mammary gland tumors in dogs are very common in clinical practice. Betulinic acid is currently a compound considered to have anticancer properties in human mammary tumors through nanoemulsions. In this study, the use of betulinic acid nanoemulsions in two female dogs diagnosed with malignant mammary tumors (MMTs) and three with benign mammary tumors (BMTs) was assessed. The responses after 30 days of daily treatment were evaluated. In one of the female dogs with MMTs treated with the nanoemulsion, the tumor size was reduced by approximately 38%, while in the BMTs of female dogs, the nanoemulsion reduced the size of the tumors by 25.3%. Oral administration of the betulinic acid nanoemulsion reduced the size of canine mammary tumors. Experimental studies are needed to further evaluate this preparation.

## 1. Introduction

The presence of mammary tumors in female dogs is frequently reported and represents a major clinical problem. Generally, approximately 50% of tumors found in dogs are malignant, the most common being tubular carcinoma, followed by papillary carcinoma, solid carcinoma, complex carcinoma, and sarcoma. In the case of benign tumors, the most frequently reported are fibroadenomas, ductal papilloma, benign mixed tumors, and simple adenomas [1]. The development of treatments to protect the health of pets, facilitate oral administration, reduce pain, and decrease adverse effects has become a priority for veterinarians [2]. Products enclosed in nanoparticles have been created and employed to prepare nanomedicines. The release of drugs through nanostructured systems (sizes less than 1000 nm) has allowed the use of pharmacotherapy in veterinary medicine to have significant advantages in administering antibiotics, antifungals, antivirals, anesthetics, antiparasitics, vaccines, and antineoplastic drugs in animals [3]. It has been reported that the effect of nanoemulsions in water has several biological activities, including antioxidant, anti-inflammatory, and anticancer properties [4]. Betulinic acid (BA) is a compound to which anticancer properties have been attributed in mammary tumors [5]. Betulinic acid is a lupane-type pentacyclic triterpenoid derived from the methanol or ethanol extraction of plant sources. It is a natural compound that can also be obtained by chemical synthesis or microbial biosynthesis [6]. However, its bioavailability when administered orally is extremely low, mainly due to the biological barriers of the digestive tract and especially to its poor solubility in water. Because of this, it is necessary to encapsulate the compounds using suitable drug delivery systems, such as nanoemulsions, which allow for increased absorption and allow them to be transported to the target tissue or organ [7]. Consequently, the objective of this work was to use a bioactive compound, betulinic acid, encapsulated in nanoemulsions as an antineoplastic treatment in mammary tumors in female dogs.

## 2. Case Description

Owners of domestic dogs with visible masses in the mammary gland attended the Hospital Veterinario de Pequeñas Especies of the Facultad de Medicina Veterinaria y Zootecnia of the Universidad Veracruzana in the city of Veracruz, Mexico. With the informed consent of the owners, five female dogs (Table 1) with a clinical history of masses in the mammary gland and whose owners were unwilling to undergo surgery as a first treatment option, underwent an individual general physical examination, as well as pre-surgical laboratory studies, including blood count and serum biochemistry profile (Table 2). All parameters showed results within normal ranges. Digital X-ray studies of the thorax and abdominal ultrasound were performed prior to treatment with nanoemulsions to rule out organ metastasis.

**Figure 1:**
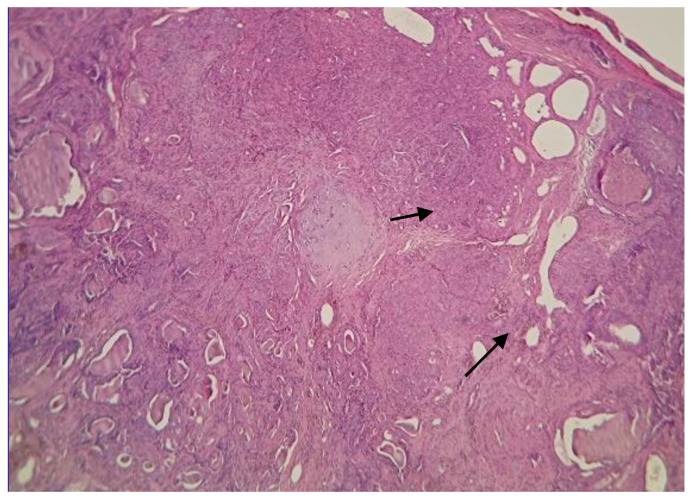
Biopsy sample dyed with H&E. Newly formed tissue was observed, composed of spindle-shaped cells forming intersecting bundles surrounding numerous tubules, some of which are irregular. Arrows indicate tubules are lined by cuboidal to slightly pleomorphic epithelial cells.

Once the laboratory results were obtained, an incisional biopsy of the tumor was taken with a punch, after anesthesia with tiletamine/zolazepam was applied at a dose of 3 mg/kg body weight intravenously, followed by propofol at a dose of 4 mg/kg for induction and inhalational anesthesia for maintenance with 1.5% sevoflurane. For pain management, meloxicam at a dose of 0.2 mg/kg and buprenorphine at 20 µg/kg were employed. Cephalexin at a dose of 22 mg/kg was used as a postoperative antibiotic. The biopsy samples were preserved in 10% formalin and sent to the histopathology laboratory to be analyzed and classified by a certified pathologist (Table 3; Figure 1). After histopathological classification of the masses of the five patients, an alternative experimental treatment to chemotherapeutics was established based on BA nanoemulsions that were composed of an oily and an aqueous phase, developed by our group. The nanoemulsions were prepared according to the method reported by Agame et al. [8], with some modifications. The following formulation was used: for the oily phase, 5 g of MCT (medium chain triglycerides) oil and 0.25 mg of BA were mixed. For the aqueous phase, 25 g of glycerol, 60 g of distilled water, and 10 g of PC (phosphatidylcholine) were dispersed. After weighing the oily and aqueous phase components, they were placed in an Aquawave 9376 ultrasonic bath (Barnstead/Labline, Waltham, MA, USA) for 5 min at 45 °C; then, to convert it into a coarse droplet nanoemulsion, an Ultra-Turrax^®^ T-25 homogenizer (IKA Works, Inc., Wilmington, NC, USA) was used at 18,000 rpm for 3 cycles of 1 min. To produce a nanometer droplet size, a 20 kHz Branson S-450D ultrasonicator (Emerson Electric Co., St. Louis, MO, USA) was used at 20% amplitude for four cycles of 1 min. Finally, particle size and the polydispersity index (PDI) were measured with a Zetasizer Nano-ZS90 (Malvern Instruments, Worcestershire, UK).

The particle size of the nanoemulsions was monitored for one week using the Zetasizer program to evaluate stability. The nanoemulsions began with an initial particle size of 245 nm and finished at 315 nm, after being stored for 6 days at 8 °C.

**Figure 2:**
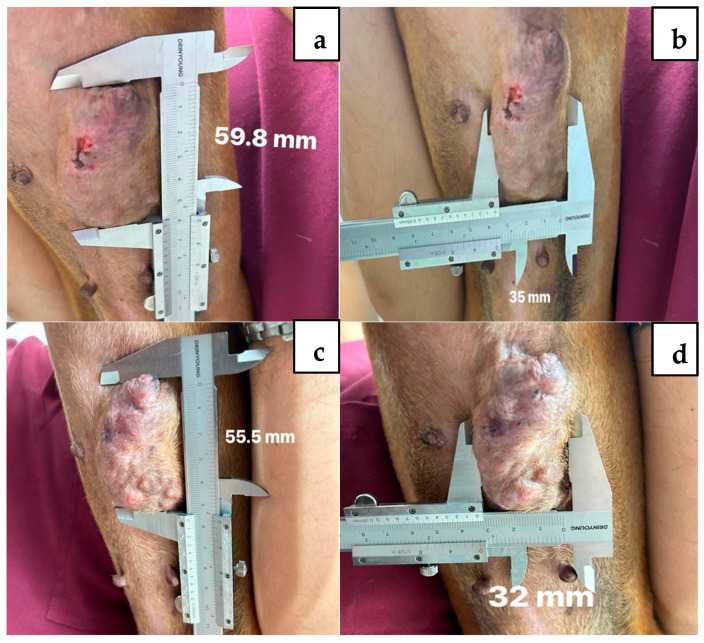
Diagnosed patient with complex mammary adenoma and decreased size after application of the nanoemulsion. Letters (**a**,**b**) are before treatment, and (**c**,**d**) are after treatment.

After preparing the nanoemulsions, oral treatment with betulinic acid nanoemulsion was administered at a dose of 5 mg/kg of body weight every 24 h for 4 weeks, with weekly evaluations of tumor size using a vernier caliper and general physical examinations of each patient (Table 4). For the evaluation of tumor response or disease progression, RECIST (Response Evaluation Criteria in Solid Tumors) criteria were used, based on tumor measurements taken before, during, and after treatment (Figure 2). There were no post-treatment changes in the evaluation of liver and kidney function values after treatment with the nanoemulsions.

## 3. Discussion

Chemotherapies have been shown to be ineffective in highly malignant mammary tumors. In a recent study of metronomic chemotherapy, NSAIDs in a grade III solid mammary carcinoma were observed [9].

One of the dogs diagnosed with a benign tumor after treatment with BA, according to RECIST, showed a favorable partial response with a tumor remission of at least 30%. At an experimental level, a synthetic BA derivative has been used to act on cancer cells in vitro. It was reported that a dose of 10 mg/mL intralesional showed excellent clinical response, including a complete remission of 5/5 animals treated with malignant neoplasms (Canine cell lines used were: melanoma, mastocytoma, osteosarcoma, fibroma and adenocarcinoma) [10]. It has been shown that BA could be designated as an anticancer drug since it has already been reported that it also inhibits the growth of cell lines of human melanoma [11]. In this study, from those dogs diagnosed with malignant tumors, one of the patients showed a 38% remission of the width of the mass, considered a partial response, and the other patient was rated as stable disease because it did not display an increase greater than 20% of the lesion. Perhaps the fact that in the study by Willmann et al. [10] a derivative was used instead of BA, in addition to being applied intra-lesionally, was probably the reason why a complete remission was found, contrary to what we observed in our study.

Tumor formation is caused by excessive proliferation and differentiation of cells or by alterations in apoptosis. Therefore, inducing apoptosis of tumor cells is a useful treatment against cancer. For the above, Zhao et al. [12] used BA and betulin in canine cancer cell lines, specifically canine lymphoma cell lines. They demonstrated effects through the inhibition of topoisomerase expression and the alteration of protein expression in related signaling pathways, concluding that they induce apoptosis and cell cycle arrest through different mechanisms in different types of tumor cells. All of the above were performed in vitro, and the authors suggest further research into these mechanisms.

Selective cytotoxicity against tumor cells and its favorable therapeutic index have also been reported, but doses of up to 500 mg/kg are discussed where no adverse effects were found, suggesting that BA could be a very promising new chemotherapeutic agent for the treatment of cancer in human medicine [13]. In animals, elevation of ALT has been reported at doses of 30 mg kg [5]. In this study, a dose of 5 mg/kg of body weight was used; hence, we would recommend further research efforts to increase the dose for longer periods to possibly observe a better remission of tumors in dogs.

## 4. Conclusions

The administration of betulinic acid nanoemulsions in accordance with the tumor evaluation criteria demonstrated that it had an effect on the reduction of mammary tumors in five dogs. It is suggested that this study should be carried out with a larger number of cases, for longer periods of exposure, and with a higher content of betulinic acid, in order to produce more conclusive data.

## Figures and Tables

**Table 1:** Household female dogs admitted to this study.

Dog	Breed	Age (Years)
1	Chihuahua	8
2	Chihuahua	10
3	Dachshund	6
4	Dachshund	10
5	Mixed	12

**Table 2:** Hematology, renal, and hepatic profile of female dogs with mammary tumors.

	Females					
	RV *	1	2	3	4	5
Red blood cells, 10^3^/mm^3^	3.3–7.8	7.6	5.3	5.9	6.4	5.3
Hemoglobin, g/dL	14.7–21.2	19.6	12.3	13.2	15.9	11.9
Hematocrit, %	44.2–62.8	58.2	36.4	40.1	47.2	36.2
GMT, fl	60–77	76.2	68.5	67.9	74.2	68.2
MHC, pg	19.0–25.9	25.7	23.1	22.3	25.0	22.5
MHCG, g/dL	31–36	33.6	33.8	32.9	33.7	32.9
Platelets, ×10^3^/mm^3^	200–500	223	233	141	226	411
WBC, ×10^3^/mm^3^	7.9–17.5	7.3	7.9	10.7	8.6	1.4
Glucose, mg/dL	77–120	78.1	74.2	52.0	61.0	61.2
Urea, mg/dL	17.6–51.8	32.8	24.7	33.9	41.6	35.3
BUN, mg/dL	7–23	15.3	11.5	15.8	19.4	16.5
Creatinine, mg/dL	0.6–1.2	0.8	0.6	0.7	1.0	0.9
Urea/Creatinine, %	>20–<10	18.9	19.5	22.9	16.2	18.9
Total cholesterol, mg/dL	129–331	177	265	158	169	236
Triglycerides, mg/dL	36–135	71.5	86.3	67.6	78.6	66.2
Uric acid, mg/dL	0.5–1.4	0.9	1.0	0.8	0.9	0.9
Direct bilirubin mg/dL	0.1–0.19	0.21	0.15	0.28	0.24	0.16
Indirect bilirubin, mg/dL	0–0.8	0.38	0.28	0.42	0.38	0.32
Total bilirubin, mg/dL	0.2–0.8	0.62	0.43	0.70	0.62	0.48
TGO, U/L	14–42	36.7	33.2	40.2	43.5	38.4
TGP, U/L	15–52	52.3	47.1	41.4	41.9	73.3
Alkaline phosphatase, U/L	20–70	90.9	141.7	44.6	56.2	131.3
γ-glutamyltranspeptidase, U/L	1.0–9.7	4.0	4.0	3.1	4.1	2.8
Total protein, g/dL	5.4–7.2	8.4	9.2	8.6	8.0	8.3
Serum albumin, g/dL	2.6–3.6	4.4	4.1	3.5	4.3	3.1
Globulin, g/dL	2.3–4.4	4.0	5.0	5.1	3.7	5.2

* Reference values.

**Table 3:** Classification of adenomas and carcinomas by histopathology.

Female	Tumor Classification
1	Complex mammary adenoma ^1^ (CMA)
2	Tubular mammary carcinoma ^2^ grade II (TBC II)
3	Complex mammary adenoma (CMA)
4	Complex mammary adenoma (CMA)
5	Tubular mammary carcinoma (TBC I)

^1^ Adenoma: tumor that is not cancerous. It starts in cells that look like glands in the epithelial tissue (thin layer of tissue that covers organs, glands, and other structures inside the body). ^2^ Cancer that forms in the glandular tissue lining certain internal organs. Most cancers of the breast, lung, esophagus, stomach, colon, rectum, pancreas, prostate, and uterus are adenocarcinomas. Also called malignant adenoma, adenoid carcinoma, adenomatous carcinoma, and glandular carcinoma [National Cancer Institute].

**Table 4:** Changes in size of mammary tumors in females under treatment for thirty days with BA nanoemulsion.

	Week of Treatment					Percentage Change	
	1	2	3	4	5	mm	%
**Female 1 (CMA)**							
**Length, mm**	8.6	8.0	6.9	6.6	6.6	−2.0	−23.0
**Width, mm**	10.8	9.0	9.0	8.8	8.6	−2.2	−20.3
**Female 2 (TBC II)**							
**Length, mm**	24.0	24.0	21.2	20.0	19.5	−4.5	−18.7
**Width, mm**	24.3	23.7	22.0	21.1	19.5	−4.8	−20.0
**Female 3 (CMA)**							
**Length, mm**	12.5	10.4	9.5	8.0	7.0	−5.5	−44.0
**Width, mm**	17.0	13.9	10.9	8.4	7.9	−9.1	−53.5
**Female 4 (CMA)**							
**Length, mm**	35.0	32.0	31.2	30.4	29.8	−5.2	−14.8
**Width, mm**	59.8	55.5	55.7	54.4	50.9	−8.9	−14.8
**Female 5 (TBC I)**							
**Length, mm**	24.4	22.2	19.9	18.2	15.0	−9.4	−38.5
**Width, mm**	51.7	50.1	49.9	49.7	48.7	−3.0	−5.8
**Average**							
**Length, mm**	20.9	19.3	17.7	16.6	15.5	−5.3	−27.8
**Width, mm**	32.7	30.4	29.5	28.4	27.1	−5.6	−22.8
**Average, mm**	26.8	24.8	23.6	22.5	21.3	−5.4	−25.3

CMA, Complex mammary adenoma; TBC II, Tubular breast carcinoma grade II; TBC I, Tubular breast carcinoma.

## Data Availability

The information published in this study is available on request from the corresponding author.
